# SPAG5 Is Involved in Human Gliomagenesis Through the Regulation of Cell Proliferation and Apoptosis

**DOI:** 10.3389/fonc.2021.673780

**Published:** 2021-11-02

**Authors:** Chunhong Wang, Haiyang Su, Rui Cheng, Hongming Ji

**Affiliations:** Department of Neurosurgery, Shanxi Medical University Shanxi Provincial People’s Hospital, Taiyuan, China

**Keywords:** SPAG5, glioma, proliferation, apoptosis, migration and invasion

## Abstract

**Background:**

Glioma is the most frequent malignant primary brain tumor in adults.

**Objective:**

To explore the role of sperm-associated antigen 5 (SPAG5) in glioma.

**Methods:**

The association between SPAG5 expression and clinical features was investigated based on The Cancer Genome Atlas (TCGA) datasets. The function of SPAG5 in glioma was analyzed using U87 and U251 cells. Knockdown glioma cells were constructed by shRNA interference. qRT-PCR and Western blotting were used to measure the expression of SPAG5 and Cadherin 2 (CDH2). Cell proliferation and apoptosis were measured by 3-(4,5-dimethylthiazol-2-yl)-2,5-diphenyltetrazolium bromide (MTT) assay, caspase 3/7 assay, and high-content screening (HCS) proliferation analysis and colony formation assay. Transwell assays and wound-healing assays were used to investigate cell migration and invasion.

**Results:**

The increased expression of SPAG5 was correlated with poor outcomes in glioma patients. Knocking down SPAG5 could inhibit the proliferation and colony formation and promoted the apoptosis of glioma cells. Knocking down SPAG5 could also inhibit cell migration and invasion and the expression of CDH2. Overexpression of CDH2 with SPAG5 depletion could restore the proliferation and inhibit the apoptosis of glioma cells, which also promoted cell migration and invasion.

**Conclusions:**

SPAG5 is a promising prognostic factor and potential therapeutic target for clinical intervention in glioma.

## Introduction

Glioma is a neuroectodermal tumor arising from glial or precursor cells (1), which represents one of the most frequent malignant neoplasm in the central nervous system (2). Studies demonstrated that glioma accounts for about 75% of primary malignant brain tumors in adults (3, 4). Despite current advances in the therapy of glioma, the overall 5-year survival rate of glioma patients undergoing comprehensive treatments, including surgical resection, adjuvant radiotherapy, and chemotherapy, is disappointingly low (5). Particularly, most of the glioma patients with high grade succumb to this disease within 2 years of diagnosis (6). Therefore, it poses great challenges to understand the potential molecular pathogenesis of glioma, to identify novel prognostic molecular markers, and to develop new therapeutic strategies (7).

Sperm-associated antigen 5 (SPAG5, also called astrin and hMAP126), which maps to Ch17q11.2 and codes for a mitotic spindle-associated protein (8), plays a key role in the regulatory network of mitosis by forming a molecular switch with a mass of protein partners (9). During mitosis, SPAG5 could interact with many proteins, such as CLASP1, astrin, and Kif2b, to regulate the centromere–microtubule dynamics and thus promotes mitotic processes and their fidelity (10). It was reported that SPAG5 has participated in growth and progression of various tumors, which was overexpressed in breast cancer (11, 12), osteosarcoma (13), lung cancer (14), bladder urothelial carcinoma (15), prostate cancer (16), and cervical cancer (17). Thus, it is deduced that SPAG5 may also take part in the tumorigenesis and progression of glioma. However, the clinical significance of SPAG5 and its biological role in glioma remain obscure.

The epithelial-to-mesenchymal transition (EMT) is a very complex process underlying cell movement during embryonic development and morphogenesis, in which several family transcription factors form a network through many signaling pathways, allowing cancer cells to acquire invasive properties and penetrate adjacent stroma (18, 19). *In vitro*, SPAG5 silencing inhibits the EMT process of osteosarcoma cells, and SPAG5 may serve as a prognostic indicator and potential therapeutic target for patients with osteosarcoma (13). Although the significance of EMT in gliomagenesis is still unclear, it has been confirmed to be closely related to glioblastomas (GBMs) (20). Cadherin 2 (CDH2) encodes N-cadherin, which is also a hallmark of EMT. Tumor endothelial cell-derived CDH2 promotes angiogenesis and has prognostic significance for lung adenocarcinoma (21). A growing body of evidence suggests that CDH2 is closely associated with glioma.

In the present study, we intended to characterize the role of SPAG5 in gliomagenesis and explore the underlying mechanisms. Our results provided the evidence that downregulation of SPAG5 represses glioma cell proliferation and attenuates glioma cell migration and invasion *in vitro*. To further explore the regulatory mechanism of SPAG5 in glioma cells, the relationship between SPAG5 and CDH2 was also analyzed. Taken together, these data demonstrate the biological and clinical significance of SPAG5 as a potential biomarker.

## Methods

### The Cancer Genome Atlas Database Analysis

We downloaded clinical characteristics and the data of SPAG5 mRNA expression profile chip expression data from The Cancer Genome Atlas (TCGA) database (http://www.cbioportal.org), including 667 glioma specimens and 10 normal specimens. The RNA-seq level 3 data of the expression profile of these samples were downloaded and sorted and directly used for the analysis of the mRNA expression of SPAG5. For pathological analysis, the RNA-seq level 3 data from the 667 glioma specimens, which was divided into low-grade gliomas (LGGs, n = 515, WHO II and WHO III grade gliomas) and GBMs (n = 152). We use the Affy and Limma packages in the R language to standardize and T test our data and then filter according to *P* value <0.05 and |FC| ≥2. According to the median of the SPAG5 mRNA expression, the 667 glioma specimens were further divided into low SPAG5 expression group (n = 334) and high SPAG5 expression group (n = 333). The association between the mRNA expression level of SPAG5 and the overall survival time of glioma patients was then analyzed by Kaplan–Meier curves.

### Cell Culture and Transfection

Glioma cell lines (U87 and U251) were purchased from Shanghai Genechem Co., Ltd. (Shanghai, China). Cells were cultured in Dulbecco’s modified Eagle’s medium (DMEM; Corning) supplemented with 10% fetal bovine serum (FBS; Ausbian) and cultured in a 5% CO_2_ incubator at 37°C. To knock down SPAG5 and overexpress CDH2, the plasmids specifically expressing SPAG5 shRNA and CDH2 mRNA were constructed using pAdTrack-CMV plasmid (Addgene, Cambridge, MA, USA) as the vector. The plasmids expressing the mRNA or shRNA that are not targeting any known human gene were used as the negative control. The cells were transfected with shSPAG5, CDH2 mRNA, or control mRNA or shRNA by Lipofectamine 2000 (Invitrogen, CA, USA) according to the instruction.

### Quantitative Real-Time Polymerase Chain Reaction

The total RNAs were extracted with TRIzol reagent (Invitrogen, Carlsbad, CA, USA) according to the manufacturer’s protocol and reversed to cDNA (Invitrogen, CA, USA). qRT-PCR was performed, and the sequences of the PCR primers are as follows: SPAG5 F: 5′-TTGAGGCCCGTTTAGATACCA-3′ and R: 5′-GCTTTCCTTGGAGC-AATGTAGTT-3′; glyceraldehyde 3-phosphate dehydrogenase (GAPDH), F: 5’-TGACTTCAACAGCGACACCCA-3′ and R: 5’-CACCCTGTTGCTGTAGCCAAA-3′. The relative expression of SPAG5 was presented as 2^-ΔΔCt^ value.

### Western Blotting

Western blotting was carried out according to the literature description (17). The primary antibodies are as follows: rabbit polyclonal SPAG5 (1:200, Sigma-Aldrich, Germany), mouse monoclonal GAPDH (1:2,000, Santa-Cruz, CA, USA), rabbit polyclonal CDH2 (1:100, Cell Signaling Technology, MA, USA), rabbit IgG (1:2,000, Santa-Cruz, CA, USA). A horseradish peroxidase (HRP)-conjugated anti-rabbit or anti-mouse IgG antibody was used as the secondary antibody (1:2,000, Santa-Cruz, CA, USA).

### Cell Proliferation and Apoptosis Assays

The 3-(4,5-dimethylthiazol-2-yl)-2,5-diphenyltetrazolium bromide (MTT) assay was used to measure cell proliferation rate. Glioma cells (2 × 10^3^ cells/well) were seeded onto 96-well plates. Subsequently, 20 μl MTT (5 mg/ml) solution was added to each well and incubated for 4 h at 37°C. After aspirating the medium, 100 μl dimethyl sulfoxide (DMSO) was added to solubilize the formazan crystals formed by viable cells. Optical density (OD) was measured at 490 nm. The observation duration lasted for 5 days.

### Colony Formation Assay

Adherent glioma cells in the logarithmic phase were trypsinized and counted to measure viability. Then, viable cells (600 cells/well) were seeded onto each well of a six-well plate. Glioma cells were allowed to adhere and grow for 15 days. Media were replaced every 3 days. When colonies were formed, we removed media and added 1 ml 4% paraformaldehyde to each well to fix cells for 30 min and stained them with crystal violet solution (22). Finally, colonies were counted. Data gathered from three independent experiments were expressed as mean colony number ± SD.

### Apoptosis Assay

Caspase 3/7 assay was used to assess apoptosis according to the manufacturer’s instructions (Caspase-Glo^®^ 3/7 Assay, Promega Corporation, Cat. No. G8092).

### Wound-Healing Assay

The 5 × 10^4^ glioma cells were inoculated in 96-well plate. When the cells grew to 90% confluence, we scratched the bottom of the dishes across each well using a scratch tester. Cells were rinsed 2–3 times with serum-free medium and cultivated in 0.5% FBS. The wound-healing process was observed for 24 h, and photos were taken at 8 and 24 h.

### Cell Migration and Invasion Assays

The cell migration and invasion assays were carried out by Transwell kit (Corning, US) following the manufacturer’s instructions. In brief, some chambers were inserted in a new 24-well plate, and 5 × 10^3^ cells in 100 μl medium without FBS were seeded on the upper chamber in Transwell apparatus. Then, 600 μl medium with 10% FBS was added in the lower chamber. After the cells were incubated for 16 h at 37°C, the medium in chambers were removed with absorbent paper, and cells on the chambers were wiped off with a cotton swab. The cells adhering to chambers were treated with 4% paraformaldehyde for 30 min to fix and stained with Giemsa solution and counted and visualized under a microscope in nine random fields (×200). The process of the invasion assay was similar to the cell migration experiment, except that the Transwell membrane was precoated with Matrigel basement membrane and the cells were cultured for 18 h at 37°C. The cell count method was the same as the cell migration assay.

### High-Content Screening Proliferation Analysis

Cells in the logarithmic growth phase were trypsinized and completely resuspended into cell suspension and counted. The cells (1,500 cells/well) were seeded onto each well of a 96-well plate and cultured at 37°C with 5% CO_2_. The day after planking, the number of green fluorescent cells was counted under Celigo cytometry system (Nexcelom, Beijing, China) for 5 consecutive days. Lentivirus 2000 was used to infect cells to make the glioma cells express SPAG5 and green fluorescent proteins, so as to facilitate the automatic cell count. To further explore the effect of SPAG5 knockdown, CDH2 was overexpressed. The experiment was divided into the following: NC+NC group (parental glioma cells + vector), KD+NC group (parental glioma cells + knockdown-SPAG5 + vector), and KD+OE group (parental glioma cells + knockdown-SPAG5 + overexpression-CDH2).

### Statistical Analysis

All quantified data were obtained from at least three independent experiments and analyzed using SPSS 17.0 software. Data are shown as mean ± SD. The Kaplan–Meier method was used for survival analysis. The log-rank test was used to assess differences in survival. Spearman’s method was applied in analyzing the relationship between gene expression level and clinical variables. Comparison of gene expression between groups was conducted using Mann–Whitney *U*. The differences between groups were analyzed using two-tailed Student’s *t*-test and ANOVA. Differences were considered statistically significant when *P* < 0.05.

## Results

### SPAG5 Expression Is Correlated With Prognosis

**Figure 1:**
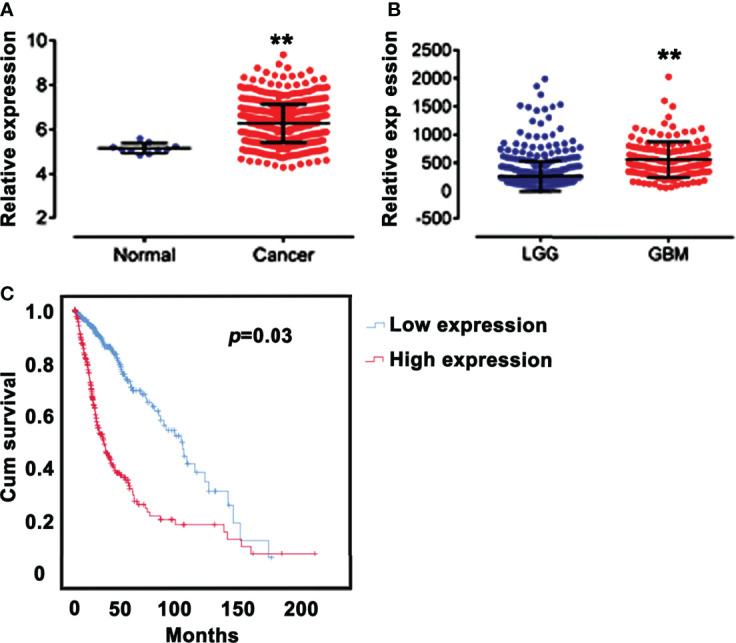
SPAG5 expression is correlated with prognosis. **(A)** Relative expression of SPAG5 in normal (n = 10) and glioma tissues (n = 667). The SPAG5 mRNA expression profile chip data including 667 glioma specimens and 10 normal specimens were all from TCGA database. vs Normal group, **P < 0.01. **(B)** Relative expression of SPAG5 in low‐grade gliomas (LGGs, n = 515) and glioblastomas (GBM, n = 152). The SPAG5 mRNA expression data of 667 glioma specimens were from TCGA database. vs LGG group, **P < 0.01. **(C)** Kaplan-Meier curves for glioma patients with low (n = 334) and high (n = 333) expression of SPAG5. According to the median, 667 glioma specimens from TCGA database were further divided into low SPAG5 expression group (n = 334) and high SPAG5 expression group (n = 333).

Analysis of the mRNA expression profiles of SPAG5 in TCGA revealed that the mRNA expression of SPAG5 was higher in glioma tissues (n = 667) than that in normal tissues (n = 10) (*P* < 0.05; **Figure 1A**). Meanwhile, the mRNA expression of SPAG5 was higher in GBMs (n = 152) than that in LGGs (n = 515) (*P* < 0.05; **Figure 1B**).

We next analyzed the relationship between the mRNA expression of SPAG5 and clinic characteristics, including age, sex, and grade (**Table 1**) in 667 glioma patients. According to the median of the SPAG5 mRNA expression, the 667 glioma specimens were further divided into low SPAG5 expression group (n = 334) and high SPAG5 expression group (n = 333). The mRNA expression levels of SPAG5 were significantly associated with overall survival time. And the high mRNA level of SPAG5 indicated poor prognosis. The expression levels of SPAG5 were not significantly associated with sex but were significantly associated with age (*P* < 0.001) and grade (*P* < 0.001). Furthermore, the overall survival between glioma patients with low (n = 334) and high (n = 333) expression of SPAG5 was compared, who were grouped according to the median of the expression of SPAG5. A Kaplan–Meier curve was obtained (**Figure 1C**). Glioma patients with low expression of SPAG5 showed a higher overall survival rate than glioma patients with high expression of SPAG5 [log-rank *P* = 0.03; hazard ratio (HR) = 3.324, CI 2.521–4.328].

**Table 1:** The relationship between SPAG5 expression level and clinic characteristics in glioma patients.

		SPAG5 expression level		Total	*P* value
		Low	High		
Age	≤46	212	128	340	<0.001
	>46	122	205	327	
Total		334	333	667	
Sex	Male	188	196	384	0.502
	Female	146	137	283	
Total		334	333	667	
Grade	LGG	317	198	515	<0.001
	GBM	17	135	152	
Total		334	333	667	

According to the median of the SPAG5 mRNA expression, the 667 glioma specimens were further divided into low SPAG5 expression group (n = 334) and high SPAG5 expression group (n = 333). LGG, low-grade glioma; GBM, glioblastoma; SPAG5, sperm-associated antigen 5.

### SPAG5 Knockdown Inhibited Cell Proliferation and Promoted Apoptosis *In Vitro*

**Figure 2:**
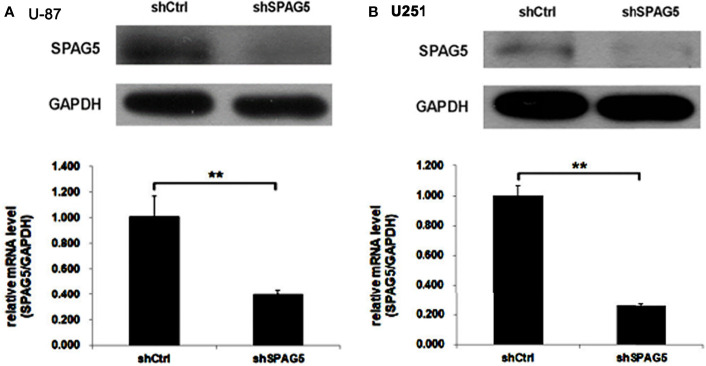
The expression of sperm-associated antigen 5 (SPAG5) in **(A)** U-87 and **(B)** U251 cells transfected with SPAG5-shRNA was measured by Western blotting and qRT-PCR, respectively. Results were expressed as mean ± SD from three independent experiments. **P < 0.01.

**Figure 3:**
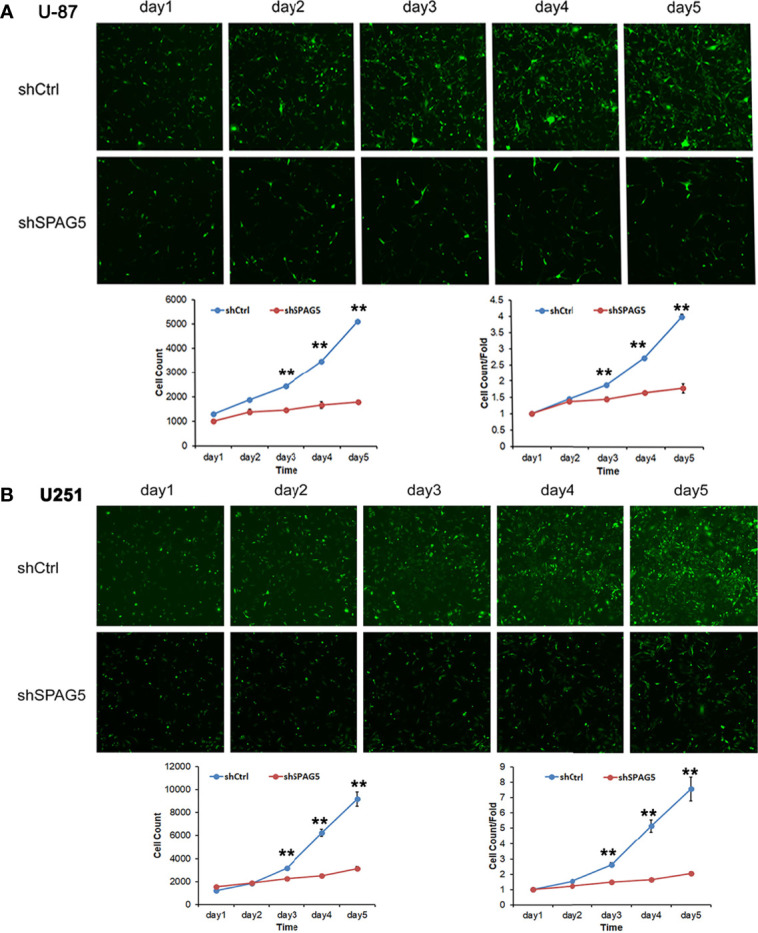
The proliferation capacities were detected by Celigo cytometry system in **(A)** U-87 cells and **(B)** U251 cells transfected with SPAG5-shRNA. Results were expressed as mean ± SD from three independent experiments. vs shCtrl group, **P < 0.01.

**Figure 4:**
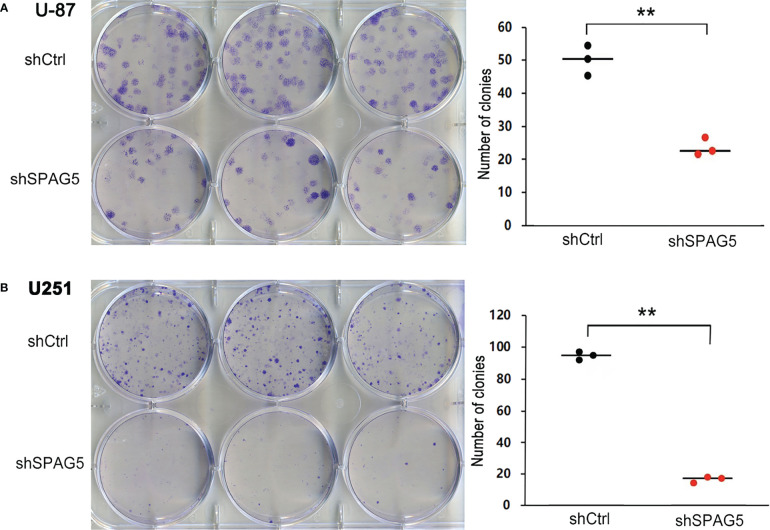
Sperm-associated antigen 5 (SPAG5) silencing reduces colony formation of **(A)** U-87 cells and **(B)** U251 cells. Bar chart showed the number of colony formation on the 15th day. Results were expressed as mean ± SD from three independent experiments. **P < 0.01.

**Figure 5:**
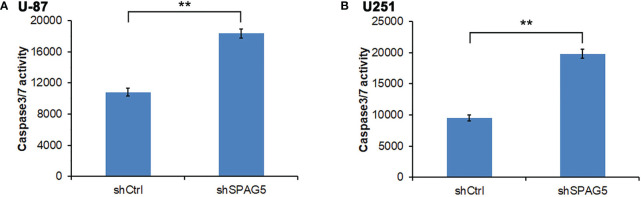
Measurement of apoptotic cells under sperm-associated antigen 5 (SPAG5) downregulation in **(A)** U-87 cells and **(B)** U251 cells. Results are to calculate the percentage of Caspase 3/7-positive cell population. Results were expressed as mean ± SD from three independent experiments. **P < 0.01.

The biological function of SPAG5 in glioma was next studied. Cell proliferation analysis was performed in the two cell lines (U87 and U251) transfected with SPAG5-shRNA. The mRNA and protein of SPAG5 in stable cell lines were examined by qRT-PCR and Western blotting. After transfection with SPAG5-shRNA, the mRNA and protein expression levels of SPAG5 in U87 and U251 cell lines were all downregulated (**Figure 2**). Knockdown of SPAG5 markedly suppressed the cell proliferation in the U87 cell lines and U251 cell lines (all *P* < 0.05; **Figure 3**). In accordance, colony formation was also significantly decreased in shSPAG5 group compared with control group on the 15th day after shRNA transfection (**Figure 4**). Caspase 3/7 assay was further carried out to assess the effect of SPAG5 on apoptosis (**Figure 5**). In the glioma cell lines (U87 and U251) transfected with SPAG5-shRNA, cell apoptosis was significantly enhanced compared with that of the normal control group. Our results revealed that SPAG5 knockdown inhibited cell proliferation and promoted apoptosis *in vitro*.

### SPAG5 Facilitates Cell Migration and Invasion

**Figure 6:**
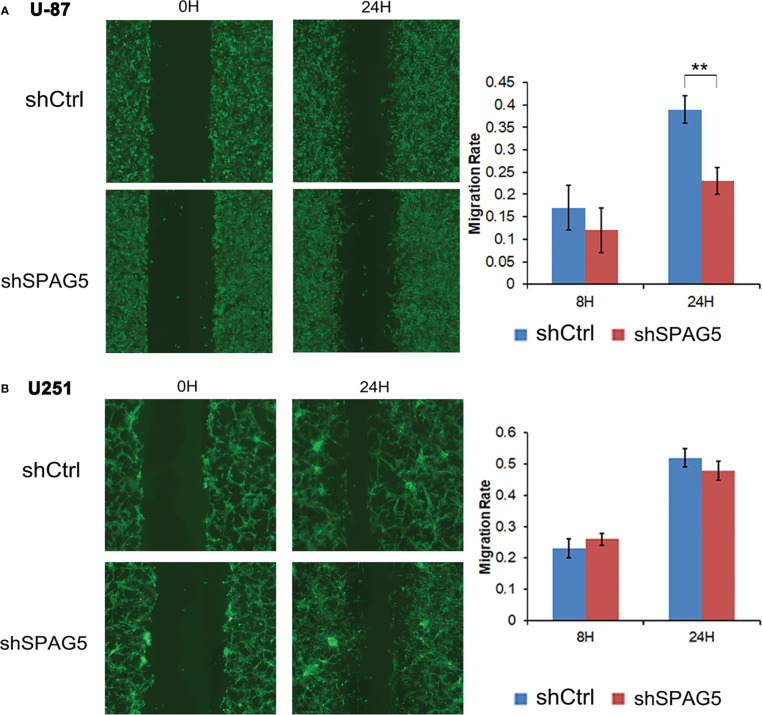
Wound healing assay showed that shRNA-sperm-associated antigen 5 (SPAG5) transfection into **(A)** U-87 cells and **(B)** U251 cells for 24 h hampered cell migrating capacity compared with that of the negative control group. Bar chart showed the relative migration ability at 8 and 24 h Results were expressed as mean ± SD from three independent experiments. **P < 0.01.

**Figure 7:**
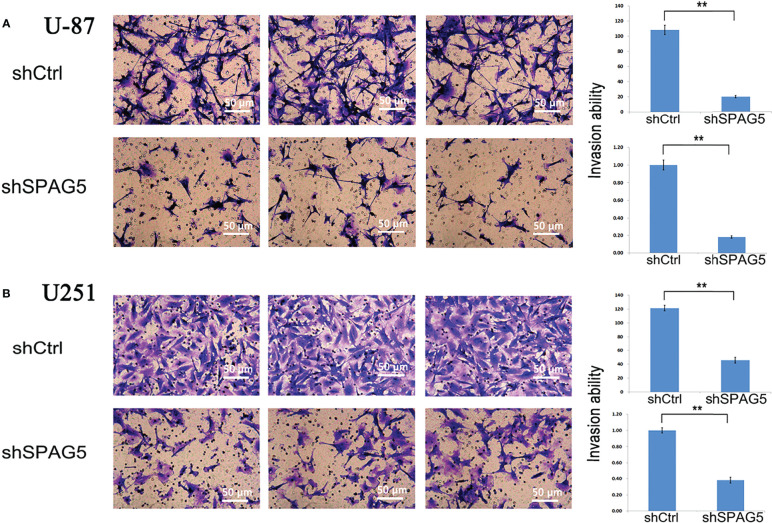
Transwell chamber assays revealed that stably suppressed sperm-associated antigen 5 (SPAG5) expression inhibited invasiveness of **(A)** U-87 cells and **(B)** U251 cells *in vitro*. Results were expressed as mean ± SD from three independent experiments. **P < 0.01.

To further analyze the effect of SPAG5 on cell migration and invasion, wound-healing assays and invasion assays were performed. The results of wound-healing assays showed that SPAG5 depletion at 24 h inhibited the migrated cells in U87 cells (*P* < 0.01; **Figure 6A**) but had no obvious effect on U251 cells (**Figure 6B**). Invasion assays demonstrated that SPAG5 enhanced the ability of cell invasion in U87 and U251 cells (**Figure 7**). The results suggested that SPAG5 might be involved in tumor progression.

### SPAG5 Knockdown Could Reduce +CDH2 Expression in Glioma Cells and Overexpression of CDH2 Could Antagonize the Effects of SPAG5 Knockdown

**Figure 8:**
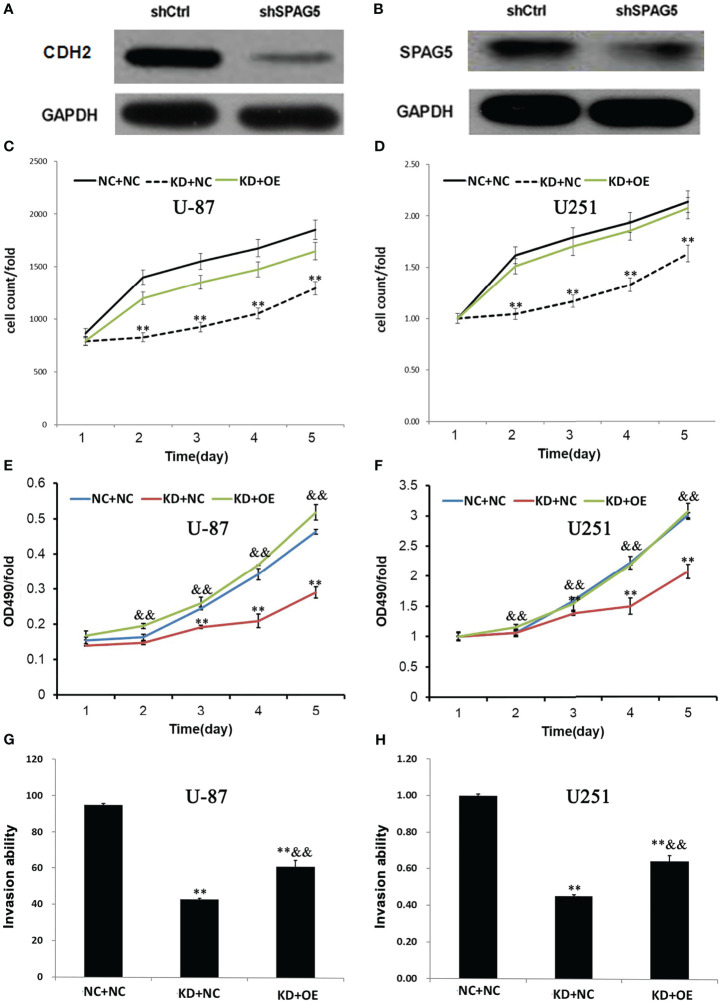
SPAG5 knockdown decreased CDH2 expression in glioma cells. The expression of **(A)** CDH2 and **(B)** SPAG5 in cells transfected with SPAG5-shRNA were measured by western blotting. The HCS proliferation screening analysis of **(C)** U-87 cells and **(D)** U251 cells. ShRNA lentivirus-infected **(E)** U-87 and **(F)** U251 cells were cultured for 5 d and used in MTT assay. The absorption rate of light at wavelength of 490 nm was compared with time in each group. OD490 reflects the number of active cells. The invasion ability of transwell transferred **(G)** U-87 and **(H)** U251 cells in each experimental group was compared with that in the control group. NC+NC: Parental glioma cells+Vector; KD+NC: Parental glioma cells+Knockdown-SPAG5+Vector; KD+OE: Parental glioma cells+Knockdown-SPAG5+overexpression-CDH2. Results were expressed as mean ± SD from three independent experiments. vs NC+NC group, **P < 0.01; vs KD+NC group, ^&&^*P* < 0.01.

To further explore the mechanisms of SPAG5 in glioma cells, several important signaling pathway molecules were examined using Western blotting. We found that the expression of CDH2 was correlated with SPAG5 knockdown (**Figures 8A, B**). The results of HCS proliferation screening analysis showed that compared with the NC group, the proliferation of the KD group was significantly reduced, which was consistent with the expectation. Compared with KD group, the expression of CDH2 gene in OE group was significantly increased (**Figures 8C, D**). MTT showed that the proliferation of KD+NC group was decreased compared with that in NC+NC group (*P* < 0.05). Compared with KD+NC group, glioma cell proliferation was increased in KD+OE group (*P* < 0.05) (**Figures 8E, F**). Compared with the NC+NC group, the Transwell transfer rate in the KD+NC group decreased (*P* < 0.05), and the Transwell transfer rate in the KD+NC group increased (*P* < 0.05) (**Figures 8G, H**). Our results revealed that SPAG5 knockdown could reduce CDH2 expression, and overexpression of CDH2 could antagonize the effects of SPAG5 knockdown in glioma cells.

## Discussion

Glioma represents the most common primary malignant cerebral tumor in adults, and especially GBM is a severe disease (7). Although glioma has a large number of studies, the precise molecular mechanisms about the disease’s development are still unclear. Additional potential markers are needed to predict glioma progression and prognosis to provide clinical significance. In this study, we reported that SPAG5 overexpression was associated with the clinical poor prognosis of glioma patients in TCGA database. Notably, SPAG5 depletion by shRNA silencing led to reduced proliferation and viability of the glioma cells. In addition, SPAG5-depleted glioma cells displayed increased apoptosis *in vitro*. In agreement, clone formation was observably decreased after SPAG5 silencing. Our data suggest that SPAG5 is an oncogene that promotes glioma by downregulating CDH2.

Ideally, identification of genes contributing to tumor genesis and progression will improve the objectivity and accuracy of tumor diagnosis and grading and will probably lead to more accurate judgments of prognosis and treatment response (1). Therefore, identification of novel diagnostic markers and therapeutic targets in glioma is urgently needed. Previous studies have fully uncovered the clinical impact of SPAG5 in breast cancer (11, 12, 23–25). Copy number aberration resulting in SPAG5 gain or amplification, as well as the high-level expression of SPAG5 transcript and protein, were accompanied by shorter overall and tumor-specific survival of patients suffering from breast cancer (25). Upregulation of SPAG5 was associated with poor prognosis in cervical cancer (17). In two independent cohorts, it was reported that HCC patients who had enhanced expression of SPAG5 frequently had a shorter survival (26, 27). SPAG5, which interacts with centrosomal protein CEP55 resulting in the phosphorylation of AKT at Ser473, promotes hepatocellular carcinoma growth *via* CEP55-mediated Phosphatidyl inositol -3- hydroxykinase (PI3K)/(protein kinase B) AKT pathway (28). SPAG5 overexpression was an independent predictor of poor prognosis in gastric cancer patients. Mechanistically, SPAG5 facilitates the progression of gastric cancer cell *via* intensifying the Wnt/β-catenin/survivin signaling *in vitro* and *in vivo (*29). This probably may be due to the fact that overexpression of SPAG5 was associated with infaust clinical factors, including poor tumor histological differentiation, large tumor volume, advanced TNM stage, lymph node metastasis status, and tumor vascular invasion. Furthermore, our findings suggested that glioma cells overexpressing SPAG5 were more aggressive. EMT-related molecules have been reported to play a key role in glioma progression.

CDH2 encodes the N-cadherin protein, and the previous study has confirmed that the expression of CDH2 in patients with high-grade glioma is higher than that in patients with LGG, and patients with high expression of CDH2 show poor prognosis (30). Our results demonstrated that deletion of the SPAG5 gene reduced the expression of CDH2 and inhibited the proliferation of glioma cells, whereas restoration of CDH2 restored the proliferation of glioma cells. This suggests that in glioma cells, the SPAG5 gene regulates tumor cell proliferation through the CDH2 signaling pathway.

Our findings imply that SPAG5 plays a role in the development of gliomas. Certain limitations of our research should be noted. Though we described that SPAG5 is related to proliferation, migration, and invasion of glioma cells at the molecular and cellular levels, the exact mechanism is not clear. Whether SPAG5 has an effect on glioma after overexpression and *in vivo* experiments are our next step need further investigation to unveil the mechanism.

## Conclusions

In short, we show that increased expression of SPAG5 in glioma was closely correlated with poor prognosis, indicating that SPAG5 serves as a promising prognostic factor in glioma. SPAG5 may represent a potential therapeutic target for the clinical intervention of glioma.

## Data Availability Statement

The raw data supporting the conclusions of this article will be made available by the authors without undue reservation.

## Author Contributions

Conception: HJ. Interpretation or analysis of data: HS and RC. Preparation of the manuscript: CW, HS, and RC. Revision for important intellectual content: CW. Supervision: CW and HJ. All authors contributed to the article and approved the submitted version.

## Funding

We thank Shanxi Province Natural Science Foundation (No. 201701D121091) for funding our study.

## Conflict of Interest

The authors declare that the research was conducted in the absence of any commercial or financial relationships that could be construed as a potential conflict of interest.

## Publisher’s Note

All claims expressed in this article are solely those of the authors and do not necessarily represent those of their affiliated organizations, or those of the publisher, the editors and the reviewers. Any product that may be evaluated in this article, or claim that may be made by its manufacturer, is not guaranteed or endorsed by the publisher.
